# The Cyclopædia of Anatomy and Physiology

**Published:** 1842-01-01

**Authors:** 


THE CYCLOPÆDIA OF ANATOMY AND PHYSIOLOGY. Edited by *Robert B. Todd*, M.D. F.R.S. &c. &c. Part XXII. London: Sherwood, and Co. 1841.

THE Number before us maintains the high character of the work. The articles Marsupialia and Monotremata by Mr. Owen, and Microscope by Dr. Carpenter, are particularly worthy of encomium.

It is inconsistent with our plan to offer any elaborate account of a work of this description. A few points are all that we can notice. The first is the—

*Period of Gestation, &c. of the Marsupialia.*

The following propositions, observes Mr. Owen, are satisfactorily established, viz. that the young of the Marsupialia are developed, primarily, as Tyson conjectured, in the true uteri or cornua uteri; but that, contrary to Tyson's opinion, they are, as compared with other Mammalia, prematurely born; and that, nevertheless, the attachment of the immature young to the nipple is essentially the same as in ordinary mammals, the young marsupial being nourished by the lacteal secretion, and its blood aerated by its own independent respiratory actions.

Such, therefore, being the condition of the problem of marsupial generation in the year 1830, there remained to be determined by exact experiment and observation the period of uterine gestation, the structure of the fœtal envelopes and appendages, the nature of the connection, if any, between the uterine fœtus and the womb, the manner of the uterine birth, and the condition and powers of the new-born young.

With a view to the solution of these questions, I applied for and obtained from the Council of the Zoological Society permission to perform the requisite experiments on the Kangaroos in the menagerie in Regent's Park. A healthy female (*Macropus major*, Shaw) was separated from the rest; she had a young one which measured about one foot two inches from the nose to the root of the tail, and which continued to return to the pouch for the purpose of sucking and for shelter. The right superior nipple was the one in use; it was nearly two inches long, and one-third of an inch in diameter; the mammary gland formed a large swelling at its base. The other three nipples were everted, and about half-an-inch in length.

A healthy full-grown male was admitted into the paddock with this female for a certain period each day, and watched, during that time, by the keeper or myself. In the course of a week the female seemed to be in a condition to excite the sexual ardour, and after a few days toying on the part of the male, she received his embrace on the 27th August, at 1 p.m. The female stood with her fore-paws off the ground, the male mounted, '*more canino*,' embracing her neck with his fore-paws, and retained his hold during a full quarter of an hour; during this period the coitus was repeated three times, and on the second occasion much fluid escaped from the vulva. The male was removed from the female in the evening of the same day, and was not afterwards admitted to her. On September the 2nd, six days after the coitus, I examined the pouch of the female, and this scrutiny was repeated every morning and evening until the birth of the young kangaroo had taken place. I select the following from the notes taken on those occasions:—

Sept. 6th.—10th day of gestation. The pouch is nearly free from its peculiar brown musky secretion. The right superior nipple retains its large size, and the young one that has left the pouch returns occasionally to suck.

Sept. 11th.—15th day of gestation. No appearance of a mammary fœtus; nipples in the same condition; the young kangaroo continues to suck and return to the pouch for shelter.

Sept. 30th.—34th day. The nipple in use by the young kangaroo (which has died) is diminished in size, and the brown secretion has begun to be formed. Qy.—Will the fœtus seize the larger nipple as the readiest, or be directed to another more proportionate to the size of its mouth?

Oct. 4th.—38th day. The keeper has observed the female putting her nose into the pouch, and licking the entry. She was examined at six in the evening; there was a slight increase of the brown secretion; the nipple formerly in use has diminished one-third in size; the other nipples indicate no appearance of approaching parturition.

Oct. 5th.—39th day. The keeper examined the pouch at seven this morning, and found there the young one attached to a nipple. On being made acquainted with this fact I repaired to the Zoological Gardens, and examined the pouch. The new-born kangaroo was attached to the left superior nipple, to the point of which it adhered pretty firmly. It measured one inch from the mouth to the root of the tail, was quite naked, and covered by a thin semitransparent vascular integument, the place of attachment of the umbilical chord was obscurely indicated by a longitudinal linear cicatrix. The fore-legs were longer and stronger than the hind ones, and the digits were provided with claws; the toes were developed on the hind legs; the body was bent forward; and the short tail tucked in between the hind legs. The little animal breathed strongly, but slowly; no direct act of sucking could be perceived. Such, after a gestation of thirty-eight days, is the condition of the new-born young of a species of Kangaroo, of which the adult, when standing erect on his hind feet and tail, can reach to the height of seven feet. The birth having taken place in the night, the mode of transference of the young to the pouch and nipple was not observed.

The hypothesis of an internal passage from the uterus to the pouch—countenanced by some imperfect anatomical observations on the course of the round ligament to the abdominal ring, and the continuation thence of the cremaster to the posterior part of the mammary gland, together with the primitive inverted condition of the nipple—is wholly refuted by more exact observations of the conditions of these parts. I was chagrined at the loss of so favourable an opportunity of determining, *ex visu*, this interesting part of the problem; for it had been my intention, if the symptoms of approaching pregnancy had been more marked, to have established a night as well as a day-watch over the female: but by placing perhaps too much reliance on the observations on the pregnant kangaroo recorded in the 9th volume of the Annales des Sciences, in which the duration of four months is assigned to the uterine gestation of this species, I had not anticipated so speedy a termination of that process as resulted from my experiment.

In order, however, to remedy, as far as might be, this omission, it occurred to me that if the young kangaroo were detached from the nipple and deposited at the bottom of the pouch, any actions of the parent, by which its original transference from the uterus to the nipple had been aided or effected, might be instinctively repeated, and thus an insight be gained into their nature. As, therefore, the experiments of Messrs. Morgan and Collie seemed too show this might be done without necessarily causing the death of the young one, I performed the experiment with the sanction and assistance of Mr. Bennett, then Secretary of the Zoological Society.

Oct. 9th.—I examined the pouch of the female, and found the young one, now four days old, evidently grown, and respiring vigorously; it adhered more firmly to the nipple than was expected, requiring a continued gentle pressure to detach it: when that took place, a minute drop of whitish fluid, a kind of serous milk, was expressed from the nipple. No blood followed, nor anything to indicate a solution of organic continuity; the extremity of the nipple was small, not swollen as in Mr. Collie's case. The young one moved its extremities vigorously. It was deposited at the bottom of the pouch, and the mother was left and then carefully watched. Soon after this was done she seemed uneasy, was often scratching the exterior of the pouch, and every now and then dilated the cavity with her two fore-paws, grasping the sides of the aperture, and pulling them in contrary directions, just as in drawing open a bag; she then inserted her muzzle pretty deeply into the pouch, moving her head about as if to lick off something from the interior, or perhaps to move the little one. She kept her nose in the pouch sometimes for half-a-minute. I never observed her to put her fore-legs, or either of them in the pouch; they were always occupied in keeping open the mouth of the pouch, while she was at work with her mouth within it. She generally concluded by licking the mouth of the pouch, and occasionally she stooped down to lick the cloaca, which she could reach with ease. When she scratched the outside of the pouch it seemed as if to push up something that was inside towards the aperture. These actions she repeated at short intervals for about an hour; she then lay down and appeared quiet. She had also lain down in the intervals of the above operation, but during that time never meddled with the pouch; when stimulated to do so by some uneasy sensation, she always rose upon her hind feet, and then inserted her muzzle alternately into the pouch and vulva. Observing the freedom with which she could reach both these parts, I was led to believe that the mode of removal of the young from the vulva to the pouch was by the mouth of the mother. Her fore-paws, in this case, would be used, not for the transport of the young, but for keeping the mouth of the pouch open for its reception, it being deposited therein by the mouth, and so held over a nipple until the mother had felt it grasping the sensitive extremity of the nipple.

This means of removal is consistent with analogy; dogs, cats, mice, all transport their young from place to place with the mouth. In the case of the kangaroo, it may be supposed that the fœtus would be held by the lips only, not the teeth, on account of its delicate consistence. Whether this theory, suggested by witnessing the actions of the mother after an artificial separation of the Marsupial fœtus, be correct, must be confirmed by actual observation. There is no internal passage from the uterus to the pouch:—the mouth of the vagina cannot be brought into contact with that of the pouch, either by muscular contraction in the living or by any force of stretching in the dead kangaroo:—as the young was proved by the result of this experiment not to have the power of itself to regain the nipple, *à fortiori* we may conclude that it could not transfer itself from the vulva to the interior of the pouch and to the apex of the nipple:—the fore-paws of the Kangaroo would not so effectually protect the tender embryo from the external air as the mouth, nor so safely ensure its passage to the pouch, notwithstanding that they are adroitly used in grasping objects, being similar, in respect of the extent and freedom of motion of the digits, to the fore-paws of the Rodents.

After the mother had rested quietly for a short time, we again examined her, but found the young one still detached, moving more vigorously than before. On an examination two days afterwards the marsupium was found empty: the young one had died and had probably been removed by the mother.

### *Nourishment of the Marsupial Embryo in Utero.*

We are at present, says Mr. Owen, ignorant of the changes that take place in the development of the ovum between the period of impregnation until about the twentieth day of uterine gestation. At this time, in the great Kangaroo (*Macropus major*,) the uterine fœtus measures eight lines from the mouth to the root of the tail: the mouth is widely open; the tongue large and protruded; the nostrils are small round apertures; the eyeball not yet wholly defended by the palpebral folds; the meatus auditorius externus is not provided with an auricle; the fore-extremities are the largest and strongest; they are each terminated by five well-marked digits; those of the hind legs are not yet developed; the cervical fold of the mucous layer or the branchial fissure is still unenclosed by the integument. The tail is two lines long, thick and strong at the commencement; impressions of the ribs are visible at the sides of the body; the membranous tube of the spinal marrow may be traced along the back between the ununited elements of the vertebral arches; posterior to the umbilical chord there is a small projecting penis, and behind that, on the same prominence, is the anus. This fœtus and its appendages were enveloped in a large chorion, puckered up into numerous folds, some of which were insinuated between folds of the vascular lining membrane of the uterus, but the greater portion was collected into a wrinkled mass. The entire ovum was removed without any opposition from a placental or villous adhesion to the uterus. The chorion was extremely thin and lacerable; and upon carefully examining its whole outer surface, no trace of villi or of vessels could be perceived. Detached portions were then placed in the field of a microscope, but without the slightest evidence of vascularity being discernible. The next membrane, whose nature and limits will be presently described, was seen extending from the umbilicus to the inner surface of the chorion, and was highly vascular. The fœtus was immediately enveloped in a transparent amnios.

On turning the chorion away from the fœtus, it was found to adhere to the vascular membrane above-mentioned, into which the umbilical stem suddenly expanded. With a slight effort, however, the two membranes could be separated from each other, without laceration, for the extent of an inch; but at this distance from the umbilicus the chorion gave way on every attempt to detach it from the internal vascular membrane, which here was plainly seen to terminate in a well-defined ridge, formed by the trunk of a bloodvessel.

When the whole of the vascular membrane was spread out, its figure appeared to have been that of a cone, of which the apex was the umbilical chord, and the base the terminal vessel above-mentioned. Three vessels could be distinguished diverging from the umbilical chord and ramifying over it. Two of these trunks contained coagulated blood, and were the immediate continuations of the terminal or marginal vessel: the third was smaller, empty, and evidently the arterial trunk. Besides the extremely numerous ramifications dispersed over this membrane, it differed from the chorion in being of a yellowish tint. The amnios was reflected from the umbilical chord, and formed, as usual, the immediate investment of the fœtus.

The umbilical chord measured two lines in length and one in diameter. It was found to contain the three vessels above-mentioned, with a small loop of intestine; and from the extremity of the latter a filamentary process was continued to the vascular membrane. The margins of the umbilicus or abdominal opening were very strong, offering much resistance to their division. On tracing the contents of the chord into the abdomen, the two larger vessels with coagulated blood were found to unite: the common trunk then passed backwards beneath the duodenum, and after being joined by the mesenteric vein, went to the under surface of the liver, where it penetrated that viscus: this was consequently an omphalo-mesenteric or vitelline vein. The artery was a branch of the mesenteric. The membrane, therefore, upon which they ramified, answered to the vitellicle, i. e. the vascular and mucous layers of the germinal membrane, which spreads over the yolk in oviparous animals, and which constitutes the umbilical vesicle of the embryo of ordinary Mammalia. The filamentary pedicle which connected this membrane to the intestine was given off near the end of the ileum, and not continued from the cœcum, the rudiment of which was very evident half a line below the origin of the pedicle.

The small intestine above the pedicle was disposed in five folds. The first from the stomach or duodenum curved over the vitelline vein, and the remaining folds were disposed around both the vitelline vessels. From the cœcum, which was given off from the returning portion of the umbilical loop of the intestine, the large intestine passed backwards to the spine, and was then bent, at a right angle, to go straight down to the anus. The stomach did not present any appearance of the sacculated structure so remarkable in the adult, but had the simple form of a carnivorous stomach. The liver consisted of two equal and symmetrically disposed lobes. The vena portæ was formed by the union of the vitelline with the mesenteric, and doubtless the other usual veins, which were, however, too small to be distinctly perceived. The diaphragm was perfectly formed.

The vena cava inferior was joined, above the diaphragm, by the left superior cava, just at its termination in a large right auricle. The ventricles of the heart were completely joined together, and bore the same proportions to each other as in the adult,—a perfection of structure which is not observed in the embryos of ordinary *Mammalia* at a corresponding period of development. The pulmonary artery and aorta were of nearly the same proportionate size as in the adult: the divisions of the pulmonary artery to the lungs were at least double the size of those observable in the embryo of a sheep three inches in length. The ductus arteriosus, on the contrary, was remarkably small. The aorta, prior to forming the descending trunk, dilated into a bulb, from which the carotid and subclavian arteries were given off.

The lungs were of equal size with the heart, being about a line in length, and nearly the same in breadth; they were of a spongy texture and of a red colour, like the veins, from the quantity of blood they contained. This precocious development of the thoracic viscera is an evident provision for the early or premature exercise of the lungs as respiratory organs in this animal: and on account of the simple condition of the alimentary canal, the chest at this period exceeds the abdomen in size.

The kidneys had the same form and situation as in the adult. The supra-renal glands were half the size of the kidneys.

The testes were situated below the kidneys, and were one-half larger than those glands, the superiority of size depending on their large epididymis, with the adherent remains of the Wolffian body. They continue within the abdomen for six weeks after uterine birth.

At a later period of uterine development, when the fœtus, measured in straight line from the mouth to the root of the tail, is ten lines in length, the urachus expands into a small allantois, of a flattened pyriform figure, and finely wrinkled external surface. This bag insinuates itself between the amnios and chorion, carrying along with it two small hypo-gastric arteries and an umbilical vein, but not establishing by their means an organized and vascular surface of the chorion by which a placental attachment is formed between the ovum and the womb. The allantois depends freely from the end of the umbilical chord, and has no connexion at any part of its circumference with the adjoining membrane. Its office is apparently that of a receptacle of urine.

The vitellicle or umbilical sac presented the same large proportionate size and vascular structure as in the first described fœtus. The chorion which enveloped this fœtus and its appended sacs was adapted to the cavity of the uterus by being disposed in innumerable folds and wrinkles. It did not adhere at any part of its surface to the uterus, but presented a modification not present in the chorion of the earlier fœtus, in being partially organized by the extension of the omphalo-mesenteric vessels upon it from the adherent vitellicle. The digits of the hind legs were distinctly formed in this embryo.

*Anatomical Condition and Development of the Marsupial Mammary Fœtus.*

The new-born fœtus of the great Kangaroo does not exceed, as we have shown, one inch in length.

By comparing ths new-born Kangaroo with a similarly sized fœtus of a sheep, we find that, although, in the Kangaroo, the ordinary laws of development have been adhered to in the more advanced condition of the anterior part of the body and corresponding extremities, yet that the brain does not present so disproportionate a size ; and the same difference is observable in the uterine fœtus of the Kangaroo, even when compared with the same sized embryo of an animal of an inferior class, as the bird. This difference, Mr. Owen apprehends, is owing to the rapidity with which the heart and lungs acquire their adult structure in the Kangaroo, whereby the passage of the purer and more nutritious blood through the foramen ovale and left auricle to the primary branches of the aorta and so to the brain is impeded. The brain, however, of the mammary fœtus, though exhibiting a low degree of development, yet is of a firmer texture than in a similarly sized fœtus of a sheep, and attains its ultimate proportion by a more gradual process of growth,

In a mammary fœtus, one inch and a half in length, the urinary bladder is largely developed, and adheres by its apex to the peritoneum, exactly opposite that part of the abdominal integument where a small linear ridge indicated the previous attachment to the umbilical chord and appendage. There are also minute but distinct traces of umbilical arteries running up the sides of the bladder to this point of attachment. As the urinary bladder becomes afterwards expanded in the abdomen, the peritoneum is gradually, as it were, drawn from this part of the abdominal parietes, forming an anterior ligament of the bladder. In a mammary fœtus of the Kangaroo about a month older than the above, there was at the superior part of this duplicature a small projecting point from the bladder, like the remains of a urachus ; but the fundus, now developed considerably above this point, was covered with a perfectly smooth layer of peritoneum ; and it is this modification, he apprehends, which led Hunter to suppose that there was no trace of urachus or umbilical arteries in the fœtuses of the *Marsupialia*. In the Sloth, the Manis, and the Armadillo, the urachus is continued in the same manner from the middle of the anterior part of the bladder, and not from the fundus.

In neither of the above fœtuses of the Kangaroo was there any corresponding trace of umbilical vein, although there was a distinct ligamentum suspensorium hepatis, formed by a duplicature of the peritoneum descending from the diaphragm to the notch lodging the gall-bladder, and not entering, as usual, the fissure to the left of that notch: the allantois is too small, and its function too limited for the preservation of any permanent trace of its peculiar vein.

The small intestines in the mammary fœtus, one inch and a half long, when compared with those of the uterine fœtus above described, were found to have acquired several additional convolutions; the fold to which the umbilical vesicle had been attached was still distinct, but now drawn in to the back of the abdomen. The cæcum was much elongated, but the colon proportionately not more developed than in the uterine fœtus; the subsequent modification, therefore, of the large intestines seems evidently destined to complete the digestion of the vegetable food.

The stomach was not sacculated, but the division between the cardiac and middle compartments was more marked than in the uterine fœtus. The liver had now advanced in its development beyond the oviparous form which it presented in the uterine fœtus, the right lobe being subdivided into three. The supra-renal glands bore the same proportionate size to the kidneys. The testes were still larger than the kidneys, and were situated below them, not having yet passed out of the abdomen: this takes place when the mammary fœtus is about three inches long from the nose to the root of the tail. The ductus arteriosus was distinct in the small mammary fœtus, but he could not perceive any trace of the thymus gland. Is this gland unnecessary on account of the precocious development of the lungs? or because of the small size and gradual growth of the brain? The latter appears the more probable condition of its absence, as in the ovoviviparous classes with small and simple brains the thymus gland is rudimental or of doubtful existence.

Notwithstanding that the new-born Kangaroo possesses greater powers of action than the same sized embryo of a sheep, and approximates more nearly in this respect to the new-born young of the rat, yet it is evidently inferior to the latter. For, although it is enabled by the muscular power of its lips to grasp and adhere firmly to the nipple, it seems to be unable to draw sustenance therefrom by its own unaided efforts. The mother, as Professor Geoffroy and Mr. Morgan have shown, is therefore provided with the peculiar adaptation of a muscle (analogous to the cremaster) to the mammary gland, for the evident purpose of injecting the milk from the nipple into the mouth of the adherent fœtus. Now it can scarcely be supposed that the fœtal efforts of suction should always be coincident with the maternal act of injection; and if at any time this should not be the case, a fatal accident might happen from the milk being forcibly injected into the larynx, unless that aperture were guarded by some special contrivance. Professor Geoffroy first described the modification by which this purpose is effected; and Mr. Hunter appears to have anticipated the necessity for such a structure, for he dissected two small mammary fœtuses of the Kangaroo for the especial purpose of showing the relation of the larynx to the posterior nares. The epiglottis and arytenoid cartilages are elongated and approximated, and the rima glottidis is thus situated at the apex of a cone-shaped larynx, which projects, as in the *Cetacea*, into the posterior nares, where it is closely embraced by the muscles of the soft palate. The air-passage is thus completely separated from the fauces, and the injected milk passes in a divided stream on either side the larynx to the œsophagus.

Thus aided and protected by modifications of structure, both in the system of the mother and its own, designed with especial reference to each other's peculiar condition, and affording, therefore, the most irrefragable evidence of creative foresight, the small offspring of the Kangaroo continues to increase, from sustenance exclusively derived from the mother, for a period of about eight months. During this period the hind legs and tail assume a great part of their adult proportions; the muzzle elongates; the external ears and eyelids are completed; the hair begins to be developed at about the sixth month. At the eighth month the young Kangaroo may be seen frequently to protrude its head from the mouth of the pouch, and to crop the grass at the same time the mother is browsing. Having thus acquired additional strength, it quits the pouch and hops at first with a feeble and vacillating gait, but continues to return to the pouch for occasional shelter and supplies of food till it has attained the weight of ten pounds. After this it will occasionally insert its head for the purpose of sucking, notwithstanding another fœtus may have been deposited in the pouch; for the latter, as we have seen, attaches itself to a different nipple from the one which had been previously in use.

*Mammary Organs.*

In the young Marsupial, as Mr. Morgan was the first to observe in the Kangaroo, the nipples are not visible, but are indicated by the orifices of a kind of cutaneous preputial sheath in which they are concealed. M. Laurent has noticed a similar condition of the nipples in a mammary fœtus of an Opossum and a Perameles. I have also observed it in the mammary fœtus of a Petaurist and Dasyure: it is doubtless, therefore, common to all Marsupials.

Once naturally protruded and the preputial sheath everted, the nipples, in the Kangaroo at least, continue external. They are longer and more slender than in other quadrupeds, and when in use generally present a terminal expansion. This part lies in a deep longitudinal fossa on the dorsum of the tongue; and the originally wide mouth of the uterine fœtus is changed to a long tubular cavity, with a terminal sub-circular or triangular aperture just large enough to admit the nipple, to which the young Marsupial thus very firmly adheres.

In the Phascogale, in which the nipples are relatively larger than usual, and of a subcompressed clavate form, the young, when grown too large to be carried in the pouch, are dragged along by the mother, if she be pursued, hanging by the nipples.

The number of nipples bears relation in the marsupial, as in the placental Mammalia, to that of the young brought forth at a birth; although from the circumstance of the produce of two gestations being for a short time suckled simultaneously, the nipples are never so few. Thus the uniparous Kangaroo has four nipples; of which the two anterior are generally those in use: the Petaurists, which bring forth two young at a birth, have also four nipples: the Thalycine has four nipples: the multiparous Virginian Opossum has thirteen nipples, six on each side and the thirteenth in the middle. In the *Didelphys Opossum* there are nine nipples, four on each side and one in the middle. The *Didelphys dorsigera* has the same number of nipples, although six is the usual number of young at a birth. In the *Phascogale penicillata* there are eight nipples arranged in a circle. The *Perameles nasuta* has the same number of nipples arranged in two slightly curved longitudinal rows; this Marsupial has three or four young at a birth.

The nipple in all the Marsupials is imperforate at the centre; the milk exudes from six to ten minute orifices arranged round the apex. It increases in size with the growth of the mammary fœtus appended to it.

The mammary gland has the same essential structure as in the ordinary Mammalia; it has no cavity or udder; its chief peculiarity arises from its being embraced by the muscle, already noticed, which has the same origin and course as the cremaster muscle in the male.

*Mammary Pouch.*

The development of the pouch is in an inverse ratio to that of the uteri and directly as that of the complicated vaginæ: thus it is rudimental in the Dorsigerous Opossum, which has the longest uteri and the simplest vaginæ: we may conclude therefore that the young undergo a greater amount of development in the womb in this and allied species.*

In the Kangaroos and Potoroos, which have the shortest uteri and longest vaginal tubes and cul-de-sac, the marsupial pouch is wide and deep. It is composed of a duplicature of the integument, of which the external fold is supported by longitudinal fasciculi of the panniculus carnosus converging below to be implanted in the symphysis pubis. The mouth of the sac is closed by a strong cutaneous sphincter muscle. The interior of the pouch is almost naked: a few hairs grow around the nipple: it is lubricated by a brown sebaceous secretion. The mouth of the pouch is directed forwards in most Marsupials: the reversed position in the Perameles and Chæropus, where the mouth is directed towards the vulva, has been already noticed. M. Laurent has made the interesting observation of the presence of a rudimental pouch in the male mammary fœtus of an Opossum: he could not discern equal traces of the nipples: that of the pouch is soon obliterated, as the scrotum increases in size.

In the male Thylacine the rudimental marsupium is retained, in the form of a broad triangular depression or shallow inverted fold of the abdominal integument, from the middle of which the peduncle of the scrotum is continued. In the female the orifice of the capacious pouch is situated nearer the posterior than the anterior boundary of that receptacle.

These extracts from Mr. Owen's paper will be perused with interest. That gentleman's contributions to the Cyclopædia are most valuable. The whole work deserves, as we have said on many previous occasions, the warm patronage of the profession.

* Is there any essential modification of the membranes of the ovum in these small Marsupials? The means of determining this question are most desirable.

